# Expanding the use of mathematical modeling in healthcare epidemiology and infection prevention and control

**DOI:** 10.1017/ice.2024.97

**Published:** 2024-09-04

**Authors:** Rebecca Grant, Michael Rubin, Mohamed Abbas, Didier Pittet, Arjun Srinivasan, John A. Jernigan, Michael Bell, Matthew Samore, Stephan Harbarth, Rachel B. Slayton

**Affiliations:** 1 Infection Control Programme and WHO Collaborating Centre for Infection Prevention and Control and Antimicrobial Resistance, Geneva University Hospitals and Faculty of Medicine, Geneva, Switzerland; 2 Division of Epidemiology, University of Utah School Medicine, Salt Lake City, UT, USA; 3 MRC Centre for Global Infectious Disease Analysis, Jameel Institute, School of Public Health, Imperial College London, London, UK; 4 Division of Healthcare Quality Promotion, U.S. Centers for Disease Control and Prevention, Atlanta, GA, USA

## Abstract

During the coronavirus disease 2019 pandemic, mathematical modeling has been widely used to understand epidemiological burden, trends, and transmission dynamics, to facilitate policy decisions, and, to a lesser extent, to evaluate infection prevention and control (IPC) measures. This review highlights the added value of using conventional epidemiology and modeling approaches to address the complexity of healthcare-associated infections (HAI) and antimicrobial resistance. It demonstrates how epidemiological surveillance data and modeling can be used to infer transmission dynamics in healthcare settings and to forecast healthcare impact, how modeling can be used to improve the validity of interpretation of epidemiological surveillance data, how modeling can be used to estimate the impact of IPC interventions, and how modeling can be used to guide IPC and antimicrobial treatment and stewardship decision-making. There are several priority areas for expanding the use of modeling in healthcare epidemiology and IPC. Importantly, modeling should be viewed as complementary to conventional healthcare epidemiological approaches, and this requires collaboration and active coordination between IPC, healthcare epidemiology, and mathematical modeling groups.

## Introduction

Mathematical modeling has been widely used across all aspects of public health response efforts during the coronavirus disease 2019 (COVID-19) pandemic to facilitate policy decisions; understand epidemiological burden, trends, and transmission dynamics; and, to a lesser extent, evaluate infection prevention and control (IPC) measures.^1–3^ Although mathematical modeling was used to inform IPC implementation prior to the COVID-19 pandemic, the use of modeling during the pandemic underscored its value as a tool for improving IPC in health care. In September 2023, 40 experts from the fields of IPC, healthcare epidemiology, and mathematical modeling convened in Geneva, Switzerland, for the 2023 IPC Think Tank, funded by the US Centers for Disease Control and Prevention. The Think Tank meeting sought to evaluate the use of mathematical modeling as a tool for healthcare epidemiology in light of the COVID-19 pandemic and highlight where mathematical modeling may augment current epidemiological approaches to IPC more broadly. In this article, we summarize the key messages and conclusions of the 2023 IPC Think Tank.

## Complementary roles of conventional epidemiology and modeling

The goals of healthcare epidemiology and IPC programs in healthcare settings include protecting patients and care providers from infectious diseases, including HAI, and limiting the spread of antimicrobial resistance. These goals are achieved through efforts such as epidemiological studies to identify risk factors for infection, surveillance for infections in the healthcare setting, and implementation and evaluation of interventions to prevent transmission of pathogens. Conventional epidemiology uses observational data to assess predictive factors and outcomes; however, these methods, at times, fall short in addressing many important questions in the fields of healthcare epidemiology and IPC. This may be, in part, because HAI and antimicrobial resistance emerge from very complex systems that are difficult, if not impossible, to directly observe or quantify and for which observational time frames are necessarily longer than ideal when rapid interventions are needed. Mathematical modeling provides a simplified representation of a complex system formalized by mathematical equations. It is an extension of conventional epidemiological analyses by which mechanistic causal processes, often not directly observed, are explicitly included. Mathematical modeling is, therefore, particularly helpful in addressing the complexity of HAI and antimicrobial resistance. Some of the similarities and differences between conventional epidemiological and mathematical modeling approaches are summarized in Table 1. Importantly, these 2 approaches are complementary rather than competing and can be used to enhance healthcare epidemiology and IPC, as described in the following examples.

### 1) Transmission pathways in healthcare settings

Understanding transmission pathways by reconstructing outbreaks (ie, who infected whom) in healthcare settings can help inform and refine infection control policies. Successful epidemiological approaches for identifying and halting nosocomial transmission rely heavily on early identification and isolation of cases and their contacts. However, conventional epidemiological approaches may not be able to provide a complete or correct picture of transmission pathways in an epidemic. This is because of the variability in the incubation period of infections due to different pathogens and variation in the extent to which transmission occurs from asymptomatic or presymptomatic persons with viral infections or from people colonized with antimicrobial-resistant bacteria.^4^ Further, key events in the mechanistic chain often cannot be directly observed in epidemiological studies; mathematical modeling approaches offer insights into unobserved events.

During the COVID-19 pandemic, the integration of viral genomic, epidemiological, and location data using a modeling framework was used to identify more accurate estimates of within-hospital transmission and transmission mechanisms and to identify individuals or groups who contribute disproportionately to transmission. In this approach, transmission is supported if the time of symptom onset and the serial interval distribution (ie, time from illness onset in a primary case to illness onset in a secondary case) of severe acute respiratory coronavirus virus 2 (SARS-CoV-2) are compatible, if location data indicate that the individuals were in the same hospital location at the time of a suspected transmission event, and if there is a high degree of relatedness between viral genomes.^4^ This approach has been used to understand SARS-CoV-2 transmission among patients and health workers in a large UK National Health Service Trust,^4^ as well as between older patients and healthcare workers in acute geriatric settings.^5^

The integration of genomic and epidemiological data to enhance understanding of nosocomial transmission was not a novel development during the COVID-19 pandemic; this method has been used to enhance understanding of methicillin-resistant *Staphylococcus aureus* (MRSA) transmission in a high transmission hospital setting^6^ and of healthcare-associated *Clostridioides difficile*.^7^ The increased availability and decreasing costs of high-throughput genomic sequencing and the development of specific software packages^8^ facilitate the integration of genomic sequencing and epidemiological data for reconstructing nosocomial transmission dynamics. This approach can be applied to the transmission of other respiratory viruses, beyond SARS-CoV-2, and potentially to multidrug-resistant organisms (MDRO) in healthcare settings.

### 2) Forecasting transmission and healthcare impact

The ability to anticipate epidemic dynamics through mathematical modeling has proven critical during the COVID-19 pandemic. The use of these models has allowed for optimizing the public health response (eg, facility closures and reopening) and allocation of healthcare demand, supplies (eg, personal protective equipment, therapeutics, and vaccines), personnel, and resources.^2,9–14^ Forecasting models have been used to provide quantitative, evaluable, and probabilistic predictions about the trajectory of SARS-CoV-2 transmission and COVID-19 impact across the near-term future.^15^ Although there may be high variation inaccuracy in forecasting COVID-19 deaths between and within individual models, there is greater accuracy using multimodel ensemble forecasts for which individual forecasts from all eligible models are combined and for which the performance exceeds most, if not all, of the models that contributed to it.^9,15^ Importantly, the accuracy of both standalone and multimodel ensemble forecasts is dependent on the quality of surveillance data. This highlights both the complementarity of conventional epidemiological and mathematical modeling approaches and the need for collaboration and active coordination between public health surveillance and mathematical modeling groups.

MDRO surveillance in Europe, for example, relies on the submission of antimicrobial susceptibility data from invasive clinical isolates. Some of the modeling approaches developed during the COVID-19 pandemic to forecast COVID-19 impact across the near-term future could be used to forecast the emergence and impact of MDRO within populations and healthcare facilities, although this is not without substantial challenges compared to forecasting acute viral infections. The major challenges to forecasting MDRO include, but are not limited to, the extent of asymptomatic carriage, susceptible and resistant strain competition and the effect of antimicrobial drug use, and longer time scales than acute viral infections.^16^ Despite these challenges, the addition of more data on colonization derived from community-based specimens and asymptomatic carriage in patients in healthcare settings could be used in a modeling framework to more accurately estimate MDRO burden (ie, deaths, hospitalizations, days of work lost, direct and indirect economic costs, and evolution of resistance).

### 3) Improving the validity of interpretation of surveillance data

Surveillance of the asymptomatic carriage of antimicrobial-resistant organisms provides data that are used to target IPC activities and to provide a deeper epidemiological understanding than is feasible with data from surveillance that includes only microbiology testing performed for clinical care. However, analyzing colonization data using conventional analytical methods has limitations, which can be resolved, at least in part, by model-based approaches. A simple example is the use of admission screening to estimate the admission prevalence of asymptomatic carriage. A more complex example is construing that a positive follow-up test in a patient with a negative admission test represents an acquisition event. In both instances, the likelihood that the observed test results are being correctly interpreted depends on the characteristics of the microbiological test (ie, false negativity and false positivity), as well as several other assumptions about the underlying dynamics. Modeling methods that explicitly represent transmission while also accounting for imperfect tests can reduce the bias associated with estimating admission prevalence and acquisition rates.^17^ Similarly, modeling approaches can provide a more accurate assessment of the probability that colonization with a resistant organism has resolved, given 2 or more consecutive negative tests.^17^ Yet another example where transmission dynamic models improve the interpretation of epidemiological data is in the analysis of the effect of colonization pressure on acquisition rates.^17,18^ All of these examples highlight the importance of using analytic methods that explicitly distinguish between observed and unobserved events.

### 4) Evaluating the impact of infection prevention and control interventions

Another area of healthcare epidemiology that mathematical modeling can support is in estimating or anticipating the impact of IPC interventions, particularly when several preventive measures are bundled together. More specifically, dynamic transmission models can be used to better understand clinical trial study design and statistical power, both to re-evaluate the true impact of a completed intervention (post hoc) and to improve the design of future clinical trials (ex ante). The use of these models can address some of the most challenging issues with IPC intervention trials, including insufficient follow-up times, outcome measurement biases, and disentangling the effects of multiple intervention components.

Two examples of post hoc analyses that illustrate how dynamic models can be used to more precisely estimate the impact of different IPC interventions studied in large clinical trials are the 2 published re-analyses of the Strategies to Reduce Transmission of Antimicrobial Resistant Bacteria in Intensive Care Units (STAR*ICU) trial^17^ and the Benefits of Universal Glove and Gown (BUGG) study.^19,20^ In the former, a Bayesian transmission modeling framework was created using data collected from the STAR*ICU trial to estimate the observed transmission and clearance rates separately for MRSA and vancomycin-resistant enterococci (VRE) while accounting for the imperfect coverage of surveillance tests. Consistent with the original trial, this work suggested no reduction of transmission for either MRSA or VRE due to the intervention, though it also revealed subtle but important differences between the pathogens regarding transmission, importation, and clearance rates. In the latter, an agent-based model calibrated and validated using data from the BUGG study was used to quantify the relative benefits of the 3 different components of the intervention, ultimately concluding that the decrease in MRSA acquisition seen in the clinical trial was likely due primarily to the barrier effects of glove and gown use. These 2 post hoc analyses show how dynamic transmission models can be used to gain deeper insights into clinical trial results and potentially direct subsequent investigations, including further evaluations of components of IPC bundles.

Mathematical models can also be very useful for improving the design of intervention trials, particularly in evaluating whether trials employ valid designs to estimate the effect of the interventions being studied accurately. Because of the nature of most hospital- or facility-based IPC intervention trials, it is difficult, complex, and costly to account for several challenging issues related to disease transmission. For instance, short hospital stays may result in unobserved acquisitions or infections that manifest postdischarge, short intervention and follow-up times may result in missing intervention effects that can build for many years before plateauing, and studies done at single centers may underestimate an intervention’s effect by not accounting for effects that may occur at other network facilities as a consequence of patient transfer. Many types of mathematical transmission models can be employed to explore the impact of accounting for these design issues on the validity of a proposed study and the extent to which it can fully estimate the effect of the intervention and its individual components.

### 5) Guiding IPC and antimicrobial treatment and stewardship decision-making

During the COVID-19 pandemic, mathematical modeling studies explored various IPC decision-making questions, including the impact of community-based non-pharmaceutical interventions,^12,21^ implementation factors for vaccination programs,^22^ and evaluation of changes to screening, vaccination, and IPC policies in nursing homes across the pandemic waves.^13,14,23–25^ Scenario modeling has been used to create and analyze different hypothetical situations and assess how they affect nosocomial transmission dynamics of SARS-CoV-2, therefore guiding implementation and, crucially, de-implementation of various IPC measures in hospitals.^25,26^

Beyond COVID-19, models have been used to assess the trade-offs of different regional containment strategies for resistant infections, helping decision-makers evaluate the trade-offs of selection of facilities and frequency of conducting point prevalence surveys^27–30^ and the cost-effectiveness of such approaches.^31^ Additionally, models have evaluated the trade-offs among prevention strategies and aided in the selection of priority facilities for recruitment into a large public health demonstration project, “Shared Healthcare Intervention to Eliminate Life-threatening Dissemination of MDROs.”^32,33^ As novel vaccines or agents designed to reduce MDRO colonization or infection make their way through the development pipeline, modeling can be used to evaluate the potential impact of these agents by accounting for not only the direct benefit to the treated individual but also the indirect population benefit resulting from prevention of transmission.^34,35^

## Expanding the use of modeling in healthcare epidemiology

The examples described above demonstrate the added value of mathematical modeling as a tool for improving understanding of HAIs and IPC efforts and facilitating public health decision-making. Table 2 highlights current priority areas for expanding the use of modeling in healthcare epidemiology and IPC. Nonetheless, there is a need to exercise some caution in this endeavor. Importantly, mathematical modeling does not replace the need for observational studies and interventional trials. Although modeling can be used when empirical data are not available, modeling cannot substitute for the absolute absence of empirical data. As such, it is increasingly important that modelers are active in the design of high-quality epidemiological studies to ensure that data needed for models are collected. Further, the expectations of modeling, as well as how modeling output should and should not be used, need to be clear. Finally, efforts to harmonize the reporting standards for modeling studies are needed to increase transparency of inputs, assumptions, uncertainties, and results. For example, the MInD-Healthcare Framework provides a standardized approach for describing and reporting agent-based models of MDRO and HAIs.^36^

It must also be underscored that traditional epidemiological studies offer invaluable insights that can enhance the accuracy and relevance of mathematical modeling in healthcare epidemiology. By providing detailed observations of the disease’s natural history, incidence rates, and risk factors, these studies offer critical data points for model calibration. For example, the detailed contact tracing efforts during the Ebola outbreak in West Africa provided data on transmission rates and incubation periods, which were crucial for developing predictive models to forecast the spread and guide intervention strategies.^37–41^ Similarly, traditional epidemiological investigations into influenza outbreaks contribute to refining mathematical models that can be used to predict the timing, peak, and intensity of influenza seasons, thereby aiding in vaccine distribution and public health preparedness.^38^ These examples underline how empirical data from traditional epidemiological studies are instrumental in informing and validating mathematical models, making them more robust and tailored to real-world scenarios in healthcare epidemiology.

## Conclusion

During the COVID-19 pandemic, there has been a rapid expansion in the use of mathematical modeling, underscoring the utility of modeling for public health and patient care. Opportunities remain to expand the use of mathematical modeling in healthcare epidemiology and IPC. Importantly, this should be seen as complementary to conventional healthcare epidemiological approaches and requires collaboration and active coordination between IPC, healthcare epidemiology, and mathematical modeling groups.

## Figures and Tables

**Table 1.** Similarities and differences between conventional epidemiological approaches and mathematical modeling approaches

	Conventional healthcare epidemiology	Mathematical modeling
Data	Collection and analysis of observed and empirical epidemiological, clinical, and laboratory data	Abstraction of a complex system, formalized by mathematical constructs; relies heavily on assumptions; parameters can be estimated directly from epidemiological data or informed by the literature or expert opinion; mechanistic causal processes are explicitly included
Techniques (examples)	Case series, case-control studies, cohort studies, randomized controlled trials	Compartmental models, network models, individual-based models, Bayesian approaches
Population	Usually hospitalized patients or persons seeking healthcare (ie, outpatient and inpatient care, residents of skilled nursing facilities); often misses community/populations up- and downstream from hospitalization	Can be used to estimate unobserved events, such as the indirect effects of secondary and tertiary transmission when empirical data are not available
Complexity	Spectrum of complexity, but may struggle to represent complex dynamic transmission or disease processes	Spectrum of complexity—often a balance between including enough information to accurately represent the system, while excluding extraneous information that limits tractability or interpretation
Feasibility	Observational studies or randomized trials are often expensive or infeasible to conceptualize and implement	More feasible, particularly when observational studies or randomized trials may be too costly, unethical, or otherwise unachievable
Time frame	Often retrospective, fixed time frame	Can be used to forecast or represent scenarios in the future
Reporting	Checklists and guidelines for reporting by study type are widely available (eg, EQUATOR network)	Increased transparency of inputs, assumptions, uncertainties, and results is needed^33^
Updates	Generally not performed	Iterative process—models can be updated as more data becomes available
Generalizability	Spectrum of generalizability, often based on study design	Spectrum of generalizability; highly specific parameterization may limit generalizability
Interpretability	Explicit interpretation of observed and measured outcomes	Can assess unmeasured outcomes and counterfactual considerations
Accessibility	Conventional statistical analysis methods and ready-to-use tools widely available	Limited access to modeling expertise; less widespread understanding of methodologies
Equity	More studies needed in low- and middle-income settings	Modeling predominantly based in high-income settings; more data generation and modeling needed in low- and middle-income settings
Informing policy decisions	Widely used to inform policy-making	Limited use to date in policy-making, likely due to limited generalizability (above); need for better communication of models and results

Note. EQUATOR, Enhancing the QUAlity and Transparency Of health Research.

**Table 2.** Priority areas for expanding the use of modeling in healthcare epidemiology and infection prevention and control

	Priority areas
Transmission modeling	- Understanding disease transmission dynamics in healthcare settings using integration of genomic, epidemiological, and location data for respiratory viruses, beyond SARS-CoV-2, and susceptible and multidrug-resistant organisms.
Forecasting	- Improved forecast accuracy for COVID-19 healthcare burden at longer horizons by all forecasting methods - Forecasting healthcare burden of infections due to multidrug-resistant organisms - Use of modeling to inform the design of surveillance systems and refine the interpretation of surveillance data
Evaluating the impact of infection prevention and control interventions	- Reanalysis of intervention clinical trial results to inform subsequent investigations - Evaluation of the effectiveness of components of infection prevention and control bundles - Use of modeling to inform the design of intervention trials
Guiding infection prevention and control decision-making	- Evaluation of the impact of novel vaccines or agents designed to reduce multidrug-resistant organism colonization or infection - Inform strategies for healthcare-associated infections, considering the potential outcomes and trade-offs associated with different interventions

Note. SARS-CoV-2, severe acute respiratory coronavirus virus 2; COVID 19, coronavirus disease 2019.
